# Pancreatic Allograft Thrombosis: Implementation of the CPAT-Grading System in a Retrospective Series of Simultaneous Pancreas-Kidney Transplantation

**DOI:** 10.3389/ti.2023.11520

**Published:** 2023-08-31

**Authors:** Palmina Petruzzo, Haixia Ye, Claudia Sardu, Olivier Rouvière, Fanny Buron, Jullien Crozon-Clauzel, Xavier Matillon, Jean Kanitakis, Emmanuel Morelon, Lionel Badet

**Affiliations:** ^1^ Department of Transplantation, Edouard Herriot Hospital, HCL, UCLB Lyon I, Lyon, France; ^2^ Department of Surgery, University of Cagliari, Cagliari, Italy; ^3^ Department of Medical Sciences and Public Health, University of Cagliari, Cagliari, Italy; ^4^ Department of Radiology, Edouard Herriot Hospital, HCL, UCLB Lyon I, Lyon, France; ^5^ Department of Anesthesiology, Edouard Herriot Hospital, HCL, Lyon, France; ^6^ Department of Dermatology, Edouard Herriot Hospital, HCL, Lyon, France

**Keywords:** simultaneous pancreas kidney transplantation, pancreas allograft thrombosis, Cambridge pancreas allograft thrombosis (CPAT) grading system, computed tomography angiography, outcome predictors

## Abstract

Pancreatic graft thrombosis (PAT) is a major surgical complication, potentially leading to graft loss. The recently proposed Cambridge Pancreas Allograft Thrombosis (CPAT) grading system provides diagnostic, prognostic and therapeutic recommendations. The aim of the present study was to retrospectively assess computed tomography angiography (CTA) examinations performed routinely in simultaneous pancreas-kidney (SPK) recipients to implement the CPAT grading system and to study its association with the recipients’ outcomes. We retrospectively studied 319 SPK transplant recipients, who underwent a routine CTA within the first 7 postoperative days. Analysis of the CTA scans revealed PAT in 215 patients (106 grade 1, 85 grade 2, 24 grade 3), while 104 showed no signs. Demographic data of the patients with and without PAT (thrombosis and non-thrombosis group) were not significantly different, except for the higher number of male donors in the thrombosis group. Pancreatic graft survival was significantly shorter in the thrombosis group. Graft loss due to PAT was significantly associated with grade 2 and 3 thrombosis, while it did not differ for recipients with grade 0 or grade 1 thrombosis. In conclusion, the CPAT grading system was successfully implemented in a large series of SPK transplant recipients and proved applicable in clinical practice.

## Graphical Abstract

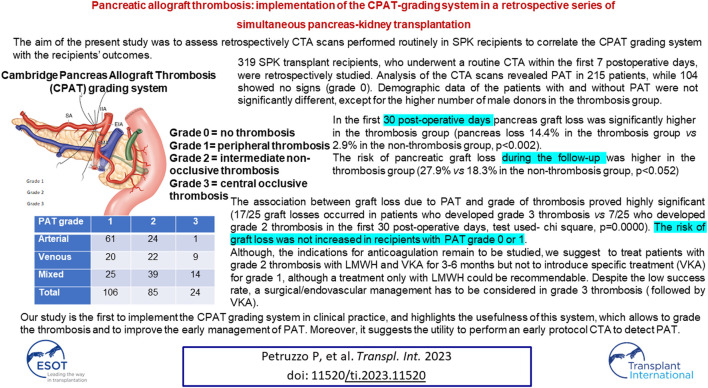

## Introduction

**FIGURE 1:**
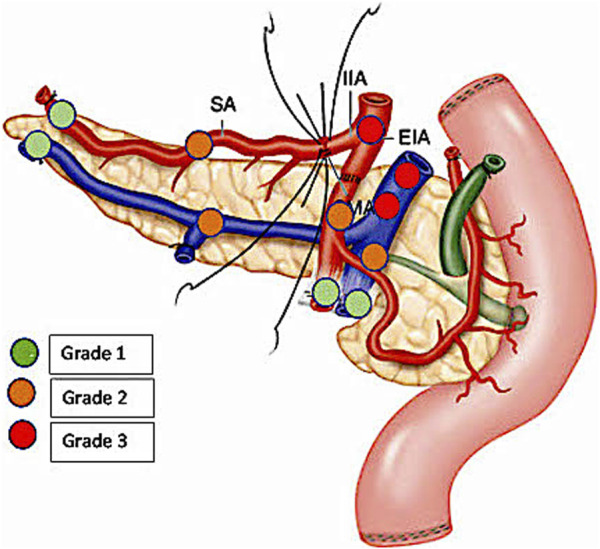
Schema showing the localization of arterial and venous allograft thrombosis (grades 1–3) on the basis of the Cambridge Pancreas Allograft Thrombosis (CPAT) grading system [10].

Pancreatic graft thrombosis (PAT) remains one of the major surgical complications and causes of graft loss in pancreatic transplantation. The reported incidence ranges from 1% to 40% [1–3] as the entity of thrombosis, ranging from partial to complete, and its extension, diagnosis, and treatment are still not well defined. In addition, partial thromboses are often underestimated, even though they are potential precursors of complete thrombosis [4, 5]. In this case, their early detection could be essential to prevent graft failure. Ultrasound and/or computed tomography angiography (CTA) are usually used to detect PAT, either routinely or when clinical symptoms develop [5–8]. The usefulness of systematic PAT detection using CTA is still debated [7–9]. Hakeem et al [10] recently proposed the Cambridge Pancreas Allograft Thrombosis (CPAT) grading system (Figure 1), which provides prognostic and therapeutic recommendations. The authors reported their experience of PAT in 103 patients who received pancreas transplantation between 2014 and 2017. In this study, CTA was performed only for biochemical/clinical reasons but not routinely. PAT was retrospectively graded on the basis of CTA to identify the risk of graft loss and outline a management algorithm through a retrospective review of these cases.

The aim of the present study was to retrospectively assess CTA examinations performed routinely in simultaneous pancreas kidney (SPK) transplantation recipients to implement the CPAT grading system [10] and to study its association with the recipients’ outcomes.

## Patients and Methods

### Design of the Study

This retrospective study included 344 patients who received for the first time a SPK transplantation between September 2005 and December 2019 at a single center.

**FIGURE 2:**
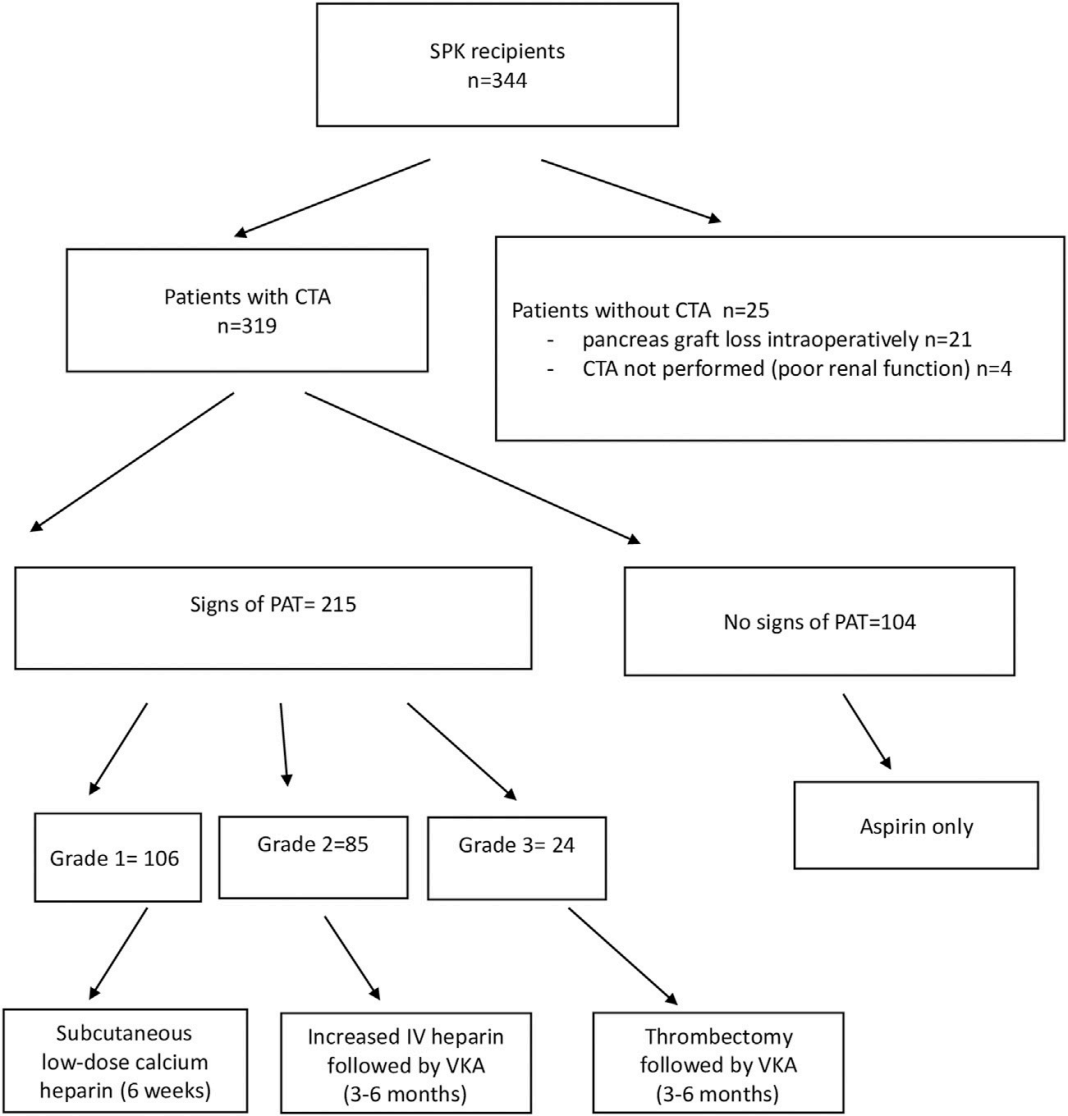
Flowchart of the study. LMWH, low molecular weight heparin; VKA, vitamin K antagonist.

In order to detect thrombosis at an early stage, 319 of the 344 patients who received SPK transplantation during the study period underwent a routine CTA of the abdomen and pelvis within the first 7 postoperative days. CTA was not performed in 25 patients because of graft loss intraoperatively or within the first hours after the transplantation (21 patients, 12 of whom lost their graft due to PAT) or because of poor renal function (four patients; Figure 2).

All CTA examinations were then retrospectively reviewed by a radiologist and a surgeon working in consensus. They assessed the presence of PAT and, when present, graded it using the classification suggested by Hakeem et al [10]:

Grade 0 = no thrombosis

Grade 1 = peripheral thrombosis

Grade 2 = intermediate non-occlusive thrombosis

Grade 3 = central occlusive thrombosis

The outcomes recorded were the incidence of PAT and its grade, its association with graft and patient survival, postoperative complications, and length of postoperative hospital stay.

Pancreas graft failure was defined as a return to insulin therapy and kidney graft failure as a return to dialysis or kidney re-transplantation. Death with a functioning graft was not considered graft failure.

### CTA Imaging Protocol

Unenhanced imaging of the abdomen was first performed, followed by an arterial phase and portal-phase contrast-enhanced acquisitions of the abdomen and the pelvis. Image analysis and data were recorded.

### Study Population

The 344 subjects included in this study had undergone SPK transplantation for the first time. They comprised patients diagnosed with type 1 diabetes mellitus since a median time of 26 years (range: 2–50 years) and end-stage renal disease, and 212 of them were on dialysis. There were 155 women and 189 men; their median age was 39 years (range: 22–58 years) and the median body mass index (BMI) was 22.5 (range: 15.8–31.2).

All the donors were brain-dead; they included 102 women and 242 men, with a median age of 31 years (range: 8–49 years). Donor cause of death was traumatic brain injury (30.5%), other trauma (21.2%), stroke (31.4%), and anoxia (12.8%). Cardiac arrest occurred in 19.8% of the donors, who spent a median time of 2.0 days (0–14) in the intensive care unit (ICU). The grafts were preserved in IGL-1 solution (80.9% of cases), Celsior solution (9.1%), University Wisconsin solution (6.9%), or Scott solution (3.1%).

### Surgical Procedure and Post-Operative Treatment

Back-bench preparation of the pancreatic graft involved removal of the spleen, ligation of all distal mesenteric vessels, and anastomosis of a donor iliac Y-graft to the graft superior mesenteric and splenic arteries in 95.3% of the donors. Portal vein lengthening was performed in 28.5% of cases.

The pancreas was placed intraperitoneally through a midline incision in the right or the left iliac fossa in 91.5% and 8.5% of recipients, respectively. Anastomosis of the portal vein was performed to the inferior vena cava or to the common iliac vein in 90.4% of the recipients, respectively. The donor iliac artery Y-graft was anastomosed to the recipients’ common iliac artery (82.3%), the external iliac artery (10.0%), or the internal iliac artery (6.8%). Exocrine drainage was performed by duodenoenterostomy (latero-lateral in 94.8% of recipients, and a Roux-en-Y duodenoenterostomy in 5.2% of them). The median cold ischemia time was 625 min (range: 330–1,162). The median anastomosis time was 31 min (range: 13–63).

The standard immunosuppression protocol included induction with antithymocyte globulins (5 mg/kg over 5 days). Maintenance immunosuppression included steroids (1 mg/kg for 3 days, progressively tapered to 5 mg/d), tacrolimus 0.05 mg/kg twice daily (trough concentration 8–12 ng/mL), and mycophenolate mofetil 1,000 mg twice daily, starting at day 0.

The patients did not receive prophylaxis with low molecular weight heparin (LMWH) but were treated with heparin (150 U/kg/d by intravenous heparin, IV) 6 h after the transplantation, before undergoing CTA. Thereafter, the patients with peripheral thrombosis received subcutaneous low-dose calcium heparin for 6 weeks post-transplantation. Intermediate PAT, not involving the arterial and/or venous donor vessels used for the reconstruction, was treated by increasing the dose of IV heparin followed by oral anticoagulant therapy (a vitamin K antagonist, VKA) for a period of 3–6 months. Complete thrombosis was treated with thrombectomy in 14 patients, followed by anticoagulant treatment, or, in 8 patients, by transplantectomy. Antiplatelet treatment (aspirin) was prescribed to all recipients (Figure 2).

The median follow-up time of the patient cohort was 5.3 years.

### Statistical Analysis

Differences between patients with or without CTA signs of PAT were assessed through the Student’s t-test for the continuous variables or the chi-square test for the categorical variables (in the latter case, when the expected values were below five, the Fisher’s exact test was used).

Univariate survival analysis was carried out through Kaplan-Meier analysis. Comparisons between survival curves were made with the log-rank test.

Multivariate survival analysis was performed with the Cox proportional hazards regression analysis. Time from transplantation to graft loss was the dependent variable. The donor’s age, gender, and BMI and the recipient’s age, gender, BMI, duration of diabetes, dialysis before transplantation, and thrombosis grade (CTA signs of PTA) were the independent variables. The independent variables that were not significantly associated and without confounding effects were removed from the model; the final model included only variables significantly associated (*p*-value <0.05).

Differences among patients who developed PAT intraoperatively or during the first post-operative hours, patients who developed PAT after the first post-operative hours but within 30 days, and patients who did not develop PAT were explored through the chi-square (for categorical variables) or the Kruskal-Wallis tests (for continuous variables with non-Gaussian distribution); in the latter case, *post hoc* analysis was performed with the Mann-Whitney test.

Analyses were performed using the SPSS V 28 software.

## Results

In total, 344 consecutive first-time SPK transplantations were performed during the study period (from February 2005 to December 2019).

At the retrospective reading by the two readers, 215 of the 319 patients had PAT on CTA, with a thrombosis grade of 1, 2, or 3 in 106, 85, and 24 patients, respectively. The 104 remaining patients had no sign of PTA (grade 0). The thromboses were diagnosed as arterial in 86 patients, venous in 51 patients, or mixed in 78 patients (Table 1).

**TABLE 1:** Grades and types of PAT.

PAT grade	1	2	3
Arterial	61	24	1
Venous	20	22	9
Mixed	25	39	14
Total	106	85	24

CTA signs of PAT were found in 215 patients, while 104 did not show such signs. These two groups of patients were compared. The patients who did not undergo CTA were not included in this comparative study (Figure 2).

There was no difference between the patients with or without PAT on CTA in terms of the donor’s age, BMI, cause of death, anoxia brain damage, cardiac arrest, and period spent in the ICU (Table 2). The only statistically significant difference was a larger proportion of donor men in the thrombosis- vs. the non-thrombosis group (73.5% vs*.* 62.1%; *p* = 0.039).

**TABLE 2:** Donor and recipient characteristics in the two groups of patients who showed (Thrombosis group) or did not show (Non-thrombosis group) CTA signs of PAT.

Donors	Non-thrombosis group	Thrombosis group	*p*-value
Gender (M/F)	64/39	158/57	*0.039*
Age (years, median)	30.0	32.0	0.558
BMI (median)	22.5	22.8	0.794
Cause of death			
Trauma	51 (49%)	115 (53.5%)	0.456
Stroke	31 (29.8)	67 (31.2%)	0.806
Anoxia	16 (15.4%)	25 (11.6%)	0.347
Suicide	17 (16.3%)	34 (15.8%)	0.903
Cardiac arrest	22 (21.2%)	43 (20%)	0.810
Days in ICU (median)	2.0	2.0	0.524
Recipients			
Gender (M/F)	56/48	123/92	0.570
Age (years, median)	38.0	40.0	0.202
BMI median	22.2	22.5	0.962
Duration of diabetes (years, median)	24.0	26.0	0.164
Dialysis	65 (62.5%)	134 (62.6%)	0.984
DSA before transplantation	5 (4.8%)	16 (7.4%)	0.374
Total HLA mismatches 1	1 (1.0%)	0 (0.0%)	0.326
Total HLA mismatches 2	3 (2.9%)	12 (5.6%)	0.401
Total HLA mismatches 3	9 (8.7%)	35 (16.3%)	0.064
Total HLA mismatches 4	30 (28.8%)	66 (30.7%)	0.735
Total HLA mismatches 5	39 (37.5%)	71 (33.0%)	0.430
Total HLA mismatches 6	22 (21.2%)	31 (14.4%)	0.130

ICU, intensive care unit.

Italic value represents the unique significant difference between the groups.

There was no difference between the two groups in terms of the recipient’s gender, age, BMI, duration of diabetes, dialysis status, and number of HLA mismatches (Table 2); preservation solution; cold ischemia time; anastomosis time; and operative procedures (Table 3).

**TABLE 3:** Procurement and operative procedures in the two groups of patients who showed (Thrombosis group) or not (Non-thrombosis group) CTA signs of PAT.

	Non-thrombosis group	Thrombosis group	*p*-value
Preservation solution			
IGL-1	77 (80.2%)	167 (81.9%)	0.732
Celsior	7 (7.3%)	20 (9.8%)	0.478
Belzer (UW)	10 (10.4%)	12 (5.9%)	0.160
Scott	2 (2.1%)	5 (2.5%)	0.844
Vessel reconstruction			
Donor iliac-Y graft	97 (93.3%)	207 (96.3%)	0.234
Splenic artery onto MSA^a^	5 (4.8%)	7 (3.3%)	0.536
Other	2 (1.9%)	1 (0.5%)	0.249
Portal vein reconstruction	29 (8.2%)	57 (26.9%)	0.813
Site of arterial anastomosis			
Common iliac artery	83 (80.6%)	177 (83.9%)	0.466
External iliac artery	12 (11.7%)	19 (9.0%)	0.461
Internal iliac artery	6 (5.8%)	14 (6.6%)	0.783
Iliac bifurcation	2 (1.9%)	1 (0.5%)	0.209
Site of venous anastomosis			
Inferior vena cava	62 (93.9%)	130 (87.8%)	0.175
Common iliac vein	3 (4.5%)	13 (8.8%)	0.256
External iliac vein	1 (1.5%)	4 (2.7%)	1.00
Superior mesenteric vein	0 (0.0%)	1 (0.7%)	1.00
Enteric drainage			
Latero-lateral	95 (99%)	194 (93.2%)	0.043
Roux-en-Y	1 (1.0%)	14 (6.8%)	0.043
Cold ischemia time (min, median)	625.0	625.5	0.785
Anastomosis time (min, median)	32.0	31.0	0.580

^a^ MSA, mesenteric superior artery.

**FIGURE 3:**
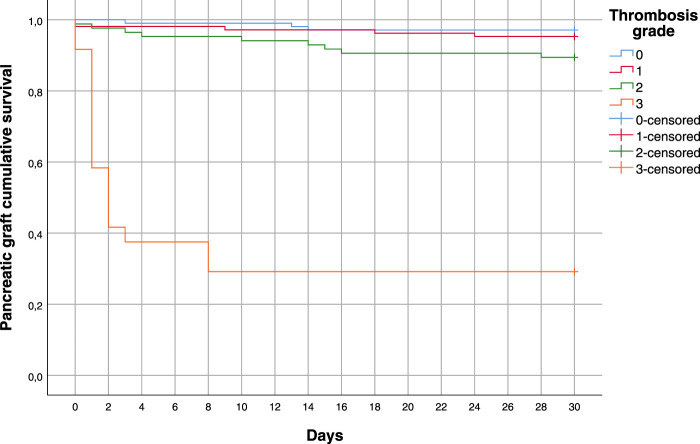
Pancreas survival within the first 30 days in patients with PAT grades 0, 1, 2, or 3, considering all causes of graft loss.

As shown in Table 4, graft survival in the first 30 post-operative days was significantly lower in the thrombosis group (pancreas loss was 14.4% in the thrombosis group vs*.* 2.9% in the non-thrombosis group, *p* < 0.002). The association between graft loss due to PAT and grade of thrombosis proved highly significant (17/25 graft losses occurred in patients with grade 3 thrombosis vs*.* 7/25 with grade 2 thrombosis in the first 30 post-operative days, *p* = 0.0000 with the chi-square test). Whatever the cause of pancreatic graft loss, it was significantly correlated to the grade of thrombosis (Figure 3). Graft losses due to PAT occurred within the first 5 post-transplant days.

**TABLE 4:** Recipient outcomes in the first 30 post-operative days in the patients who showed (Thrombosis group) or did not show (Non-thrombosis group) CTA signs of PAT.

	Non-thrombosis group	Thrombosis group	*p*-value
Pancreas loss - any cause	3 (2.9%)	31 (14.4%)	0.002
Pancreas loss - thrombosis	1 (1.0%)	24 (11.2%)	0.001
Kidney loss - any cause	2 (2.0%)	4 (1.9%)	1.000
Death	1 (1.0%)	1 (0.5%)	0.546
Days in ICU (median)	3.0	3.0	0.587
Hospitalization days (median)	23.0	22.0	0.699

During the first 30 post-operative days, there was no difference between the two groups in the number of kidney graft losses, deaths, and ICU and hospitalization days (Table 4).

There was a statistically significant difference in the number of thrombotic complications and re-interventions between the groups, (11.2% in the thrombosis group vs*.* 1.9% in the non-thrombosis group, *p* < 0.001 and 14.4% vs*.* 1%, *p* < 0.001, respectively), and a significantly higher number of transplantectomies in the thrombosis group (13% in the thrombosis group vs*.* 0% in the non-thrombosis group, *p* < 0.001) (Table 5). There was no difference between the two groups regarding the number of other surgical complications (35.6% in the non-thrombosis group vs*.* 34.9% in the thrombosis group) or surgical re-exploration (34% in the non-thrombosis group vs*.* 34.4% in the thrombosis group). There was no significant difference between the incidence of surgical complications or surgical re-explorations or graft loss due to bleeding between the thrombosis group, which received anticoagulation, and the non-thrombosis group (Table 5).

**TABLE 5:** Surgical complications and re-explorations, graft loss, and patient death during the follow-up (median follow-up 5.3 years) in the patients who showed (Thrombosis group) or did not show (Non-thrombosis group) CTA signs of PAT.

	Non-thrombosis group	Thrombosis group	*p*-value
Surgical complications	38 (36.5%)	75 (34.9%)	0.772
Thrombosis	2 (1.9%)	32 (14.9%)	<0.001
Bleeding	17 (16.3%)	24 (11.2%)	0.195
Enteric leak	8 (7.7%)	8 (3.7%)	0.128
Peritonitis	3 (2.9%)	7 (3.3%)	1.000
Small bowel obstruction	1 (1.0%)	7 (3.3%)	0.282
Eventration	3 (2.9%)	4 (1.9%)	0.687
Surgical re-exploration	35 (34.0%)	74 (44.4%)	0.939
Thrombosis	1 (1.0%)	31 (14.4%)	<0.001
Bleeding	15 (14.6%)	22 (10.2%)	0.260
Small bowel obstruction	3 (2.9%)	4 (1.9%)	0.687
Enteric leak	7 (6.8%)	10 (4.7%)	0.426
Eventration	3 (2.9%)	5 (2.3%)	0.717
Transplantectomy	0 (0.0%)	28 (13.0%)	<0.001
Peritonitis	4 (3.9%)	5 (2.3%)	0.478
Other	6 (5.8%)	4 (1.9%)	0.083
Pancreas loss	19 (18.3%)	60 (27.9%)	0.062
Thrombosis	1 (1.0%)	28 (13.0%)	<0.001
Bleeding	2 (1.9%)	2 (0.9%)	0.599
Peritonitis	5 (4.8%)	8 (3.7%)	0.764
Acute rejection	3 (2.9%)	3 (1.4%)	0.396
Chronic rejection	2 (1.9%)	5 (2.3%)	1.00
Diabetes recurrence	2 (1.9%)	5 (2.3%)	1.00
Kidney loss	10 (9.8%)	25 (11.9%)	0.581
Death	6 (5.8%)	18 (8.4%)	0.409

Two patients in the non-thrombosis group suffered from peritonitis which prompted re-intervention and PAT detection.

**FIGURE 4:**
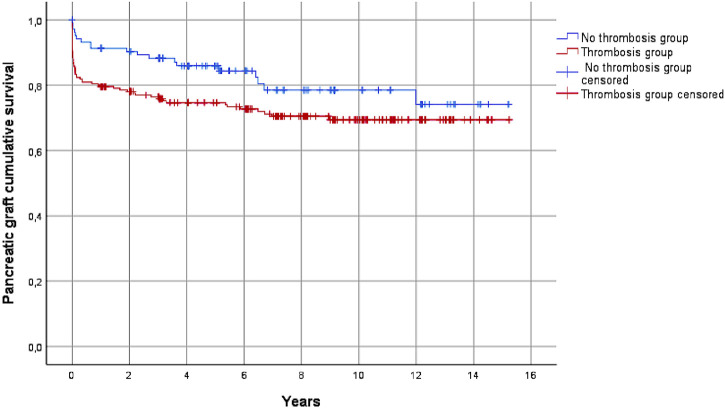
Pancreas graft survival in the thrombosis and the non-thrombosis groups during the follow-up, considering all causes of graft loss (*p* = 0.052).

As shown in Table 5, the risk of pancreatic graft loss during the follow-up was higher in the thrombosis group (27.9% vs*.* 18.3% in the non-thrombosis group, *p* < 0.052). This result was also confirmed by the Kaplan-Meier analysis (Figure 4).

The main cause of pancreatic graft loss was thrombosis (13% in the thrombosis group vs*.* 1% in the non-thrombosis group, *p* < 0.001); other causes included bleeding, peritonitis, acute and chronic rejection, and diabetes recurrence, with no significant difference between the two groups (Table 5).

**FIGURE 5:**
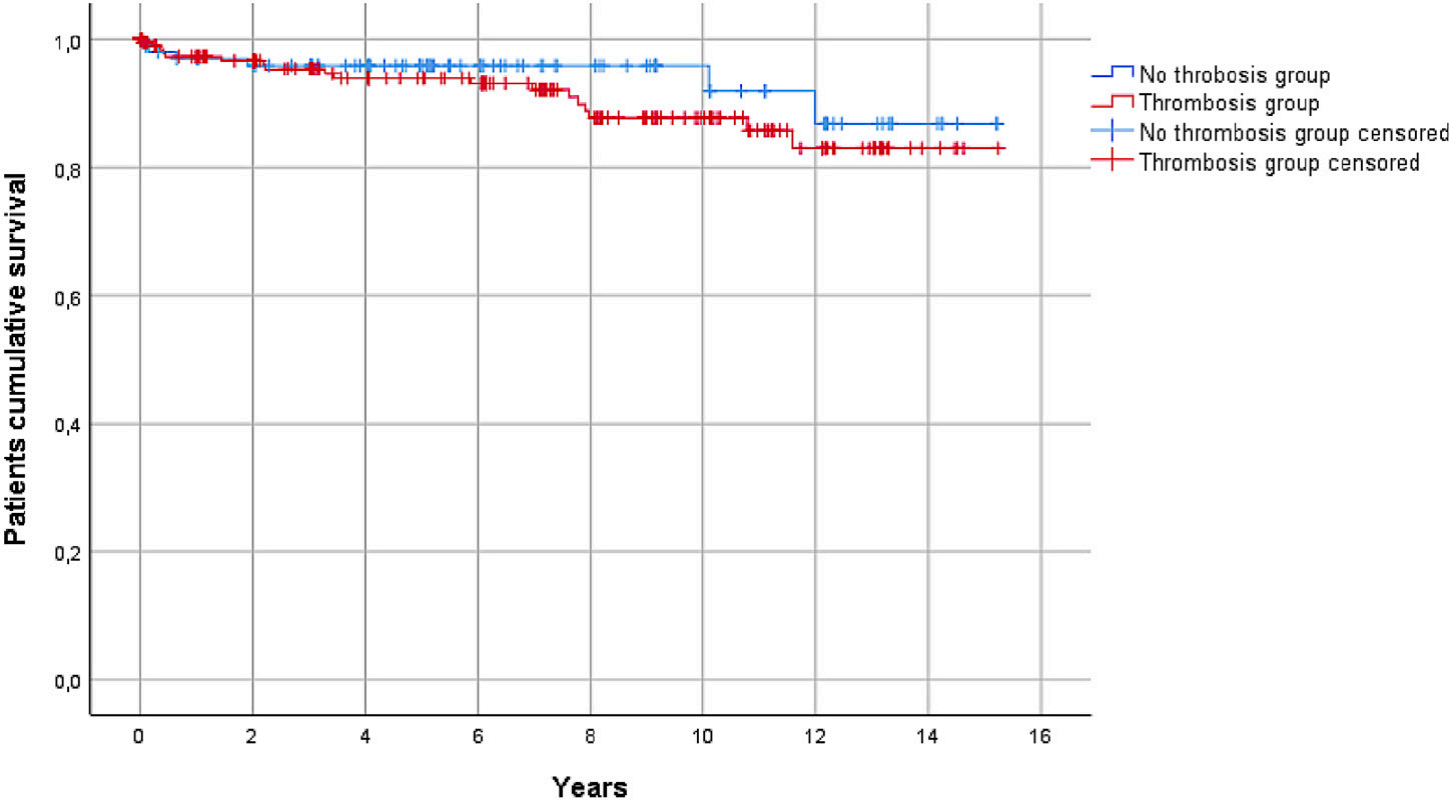
Patient survival during follow-up did not differ significantly between the thrombosis and the non-thrombosis groups (*p* = 0.347).

Patient survival was not correlated to pancreatic graft thrombosis (Figure 5).

The multivariate analysis (Cox proportional hazard model) showed that the risk of pancreatic graft loss was significantly associated with the recipient’s age, the development of hyperglycemia, hemorrhage, abdominal pain, and thrombosis grade 2 or 3, while there was no increase in the risk of graft loss in recipients with PAT grade 0 or 1 (Table 6).

**TABLE 6:** COX proportional hazard final model (including only significant associated variables).

	Hazard ratio	95% confidence intervals	*p*-value
Recipient age (years)	1.05	1.00–1.09	0.04
Hyperglycemia (yes vs. no)	2.72	1.13–6.52	0.03
Hemorrhage (yes vs. no)	2.82	1.13–7.02	0.03
Abdominal pain (yes vs. no)	4.23	1.74–10.33	0.00
Thrombosis grade 1 vs. grade 0	1.94	0.46–8.15	0.37
Thrombosis grade 2 vs. grade 0	5.18	1.37–19.63	0.02
Thrombosis grade 3 vs. grade 0	44.29	12.14–161.53	<0.01

Dependent variables: time from transplantation to graft loss during the first post-transplant 30 days. Independent variables: all the factors which can induce graft loss. All potential confounding factors were taken into account.

Only in the 12 patients who developed PAT intraoperatively or during the first post-operative hours (they were not included in the thrombosis group) there was a correlation between PAT occurrence and donors’ age and the recipients’ duration of diabetes. The Kruskal-Wallis analysis showed that the donor’s age was significantly higher in patients who developed PAT intraoperatively than in those who developed it after the first post-operative days but within 30 post-transplantation days (41 vs*.* 32 years, *p* = 0.02) or in the patients who did not develop PAT (41 vs*.* 29 years, *p* = 0.01). Similarly, the duration of the recipient’s diabetes was significantly higher in the patients who developed PAT intraoperatively than in those who developed PAT after the first post-operative hours but within 30 days (32 vs*.* 26 years, *p* = 0.01) or in the patients who did not develop PAT (32 vs*.* 24 years, *p* = 0.01).

## Discussion

This study addressed PAT that occurred following SPK transplantation in a large series of patients who underwent transplants at a single center. The study included only patients with type 1 diabetes and end-stage renal disease who received a SPK transplantation for the first time, in order to exclude additional risks of PAT.

To our knowledge, this is the first study that implemented the CPAT grading system in clinical practice after Simonis SA et al [11] assessed the applicability and the reproducibility of this system.

The large majority of the recipients (319/344, i.e., 93%) underwent systematic CTA to detect early signs of thrombosis. CTA was not performed in 25 recipients, 21 of whom had lost their pancreatic allograft intraoperatively or within the first hours after the transplantation, and 4 of whom had shown poor renal function recovery.

Although there is no consensus on when systematic CTA should be performed [8–10], we decided to perform it within the first 7 post-operative days or sooner when the patients presented signs of complications (i.e., hyperglycemia). CTA was chosen for its high specificity and sensitivity, and non-operator dependence [8, 12–14]. It was well tolerated without a significant decrease in renal function [9–11]. It was not performed only in a few patients to avoid further kidney injury. PAT was detected by CTA except in 12 patients who developed it in the operating room. Moreover, it was not diagnosed by protocol CTA in two patients, in whom PAT was detected in the operating theater during a re-operation for other causes.

In the present study, the incidence of PAT was high because all the recipients underwent CTA and all grades of thrombosis were considered, contrasting with the majority of studies where CT scans were not performed routinely in all recipients but merely in those showing graft dysfunction, or following the appearance of symptoms [8–10]. Moreover, in the study of Simonis SA et al [11], 80%–90% of the re-analyzed CT scans showed signs of thrombosis.

In the present study, the retrospective analysis of CTA showed 106 grade 1, 85 grade 2, and 24 grade 3 thromboses, which were all included in our analysis, while grade 1 thromboses were not considered in the majority of the previous studies [8–10].

The demographic data of the two groups (thrombosis and non-thrombosis) did not show significant differences, except for the higher proportion of male donors in the thrombosis group (73.5% in the thrombosis- vs. 62.1% in the non-thrombosis group) and a higher incidence of thrombosis in patients with Roux-en-Y enteric drainage, but the number of these patients is too small to be considered informative. Shahrestani S et al [15] also found that the risk of thrombosis increased by 25.6-fold in the case of male donors. Interestingly, the donor’s age and the duration of the recipient’s diabetes were significantly associated with the risk of developing PAT only in the 12 patients who developed it intraoperatively or during the first post-operative hours. These risk factors have been reported in many studies [6, 16–19], but in our study, we found a significant association between them and the occurrence of PAT only in these 12 patients.

The majority of the grade 1 thromboses were arterial (81.1%), while the thromboses graded 2 or 3 were either venous or mixed (77.1%).

In the present study, patients with grade 1 thrombosis had a favorable course (none of them lost their graft of PAT). Indeed, the survival analysis showed that the risk of graft loss was the same in recipients with grade 0 or grade 1 thrombosis; conversely, patients with PAT grades 2 or 3 were at a significantly higher risk of graft loss due to PAT (7/25 and 17/25, respectively) compared to patients with grades 0 or 1. Moreover, even though there was no significant difference between the two groups in the number of surgical complications, whatever the cause of pancreatic graft loss (including bleeding and pancreatitis), the risk was significantly associated with PAT grades 2 and 3.

Currently, no standard protocol exists that is able to consistently prevent thrombosis of the arterial or venous anastomosis sites or within the extension grafts following transplantation [9, 10, 20, 21]. In the present study, the patients did not receive prophylaxis with LMWH but were treated for a few days with IV heparin before undergoing the protocol CTA [20–23].

The retrospective review of the CTA scans and the use of the grading system allowed us to grade the thromboses and address the management of PAT. Indeed, only 7/85 (8.2%) of the patients who developed grade 2 thrombosis and 17/24 (70.8%) of those who developed grade 3 thrombosis lost their pancreatic graft. Although the indications for anticoagulation remain to be studied [7, 10, 17, 23–25], we suggest treating patients with grade 2 thrombosis with LMWH and VKA for 3–6 months but not introducing specific treatment (VKA) for grade 1. However, treatment only with LMWH in grade 1 could be recommendable. Despite the low success rate, surgical/endovascular management has to be considered in grade 3 thrombosis [26, 27] followed by VKA. Moreover, careful donor selection and prophylaxis with LMWH in preventing thrombosis could be useful. In the present study, 12 patients lost their pancreatic graft in the immediate post-operative period before performing CTA and starting any anticoagulation treatment. This group of patients is important, rendering necessary a better knowledge of donor and recipient characteristics (i.e., thrombophilia abnormalities) to identify the high-risk patients before transplantation (i.e., in the group of patients not on dialysis).

Our study has some limitations. Firstly, the study is retrospective and includes patients who underwent transplants over a long period of time by different surgeons with different experience and we have to consider that some cases of PAT might be associated with the surgical procedure. Moreover, some difficulties were experienced in the implementation of the CPAT grading system, particularly in the differentiation between grades 0 and 1, already experienced by Simonis SA et al [11].

In conclusion, the CPAT grading system was successfully implemented in a large series of SPK transplantations and showed its applicability in clinical practice. We suggest an early protocol CTA to detect PAT and a large prospective study introducing subgrouping in the CPAT system to better establish clear indications for PAT prophylaxis and treatment (28).

## Data Availability

The raw data supporting the conclusion of this article will be made available by the authors, without undue reservation.

## Abbreviations

BMI, body mass index; CPAT, Cambridge pancreas allograft thrombosis; CTA, computed tomography angiography; ICU, intensive care unit; IV, intravenous; LMWH, low molecular weight heparin; PAT, pancreas allograft thrombosis; SPK, simultaneous pancreas kidney; VKA, vitamin K antagonist.
